# Disease burden of congenital Zika virus syndrome in Brazil and its association with socioeconomic data

**DOI:** 10.1038/s41598-023-38553-4

**Published:** 2023-07-23

**Authors:** Bruna Luiza de Amorin Vilharba, Mellina Yamamura, Micael Viana de Azevedo, Wagner de Souza Fernandes, Cláudia Du Bocage Santos-Pinto, Everton Falcão de Oliveira

**Affiliations:** 1 Programa de Pós-Graduação em Doenças Infecciosas e Parasitárias, Universidade Federal de Mato Grosso do Sul, Campo Grande, MS Brasil; 2 Departamento de Enfermagem, Universidade Federal de São Carlos, São Carlos, SP Brasil; 3 Faculdade de Medicina, Universidade Federal de Mato Grosso do Sul, Campo Grande, MS Brasil; 4 Universidade Federal de Mato Grosso do Sul, Hospital Universitário Maria Aparecida Pedrossian-HUMAP-EBSERH, Campo Grande, MS Brasil

**Keywords:** Diseases, Infectious diseases, Viral infection, Public health, Epidemiology

## Abstract

Zika virus (ZIKV) infection became a global public health concern, causing an epidemic in Latin America from 2015 to 2016, when a sudden increase in cases of microcephaly and other congenital anomalies was observed. In 2016, the Centers for Disease Control and Prevention and the World Health Organization defined congenital Zika-associated syndrome (CZS) as a set of congenital anomalies seen in children born to mothers with a history of gestational Zika fever, who have microcephaly as the most prevalent clinical sign. In order to describe the magnitude of CZS in Brazil, this study estimated the burden of disease due to CZS in Brazil using the disability-adjusted life years (DALY) indicator and other frequency measures, such as incidence and mortality rate, during the years 2015–2020. The association of these indicators with socioeconomic variables was also evaluated using Spearman's correlation coefficient. Choropleth maps were used to evaluate the spatial distribution of the indicators evaluated and the spatial autocorrelation was verified by the Bivariate Moran Local Index. From 2015 to 2020, 3,591 cases of CZS were confirmed in Brazil, with an incidence of 44.03 cases per 1000 live births, and a specific mortality of 12.35 deaths per 1000 live births. A global loss of 30,027.44 DALYs was estimated from 2015 to 2020. The Northeast region had the highest values for all health indicators assessed. Spatial correlation and autocorrelation analyses showed significant associations between health and socioeconomic indicators, such as per capita income, Gini index, illiteracy rate and basic sanitation. The study allowed us to have access to all reported cases of CZS, showing us the possible situation of the disease in Brazil; therefore, we believe that our results can help in the understanding of future studies.

## Introduction

The Zika virus (ZIKV) is a flavivirus, whose main route of transmission to humans is vectorial^1^. ZIKV infection became a Public Health Emergency of International Concern when it caused an epidemic in Latin America from 2015 to 2016, when a sudden increase in cases of microcephaly and other congenital anomalies was observed in places where the epidemic was ongoing^2^. One of the most serious outcomes associated with ZIKV is congenital ZIKV syndrome (CZS)^3,4^, which is characterized by a series of congenital anomalies, such as microcephaly, hydrocephalus, the presence of intracranial calcifications, decreased brain volume, arthrogryposis and joint deformities, exaggerated primitive reflexes, hyperexcitability, hyperirritability, and ocular changes, among others^4–7^.

The ZIKV epidemic brought with it several economic and social problems, especially for the families and mothers of children with CZS^8,9^, as well as for health systems^10,11^. Moreover, numerous families reliant on the Brazilian public health system, known as the Unified Health System (*Sistema Único de Saúde*), encounter a multitude of inequities and barriers when attempting to access specialized healthcare services^8^.

Assessing the impacts caused by the emergence of ZIKV is necessary and the use health indicators that measure aspects related to the burden of the disease, including the social and economic data, is essential for it. The disability-adjusted life years (DALY) has been used to quantify the burden of various diseases in different settings and contexts^12–14^. This indicator summarizes the values of morbidity and mortality caused by disease, whether infectious or not, in a single measure^15^.

From 2015 to 2020, the Ministry of Health of Brazil received 18,228 notifications of suspected cases of CZS, of which 3,523 were confirmed^16^; thus, this is a study aiming to assess the burden of disease by CZS, especially in relation to the impacts caused by cases of premature death and years lived with congenital anomalies. Additionally, the results obtained may help in proposing public policies aimed at CZS. Based on the above, this study aimed to estimate the burden of disease due to CZS in Brazil from 2015 to 2020 and to verify the spatial autocorrelation of the DALYs for CZS. In addition, the association between morbidity, mortality, and socioeconomic indicators was also assessed.

## Methods

### Study design

This work is an ecological study that was carried out with Brazilian national data set, covering the period from 2015 to 2020.

### Study population

All confirmed cases of CZS that were reported to the Public Health Events Registry (RESP-Microcephaly) during 2015–2020 and all deaths from CZS in the same period were included in the study.

The definition of confirmed case adopted in this study is the same used by the Ministry of Health of Brazil: a set of congenital anomalies that may include visual, auditory and neuropsychomotor alterations that occur in individuals (embryos or fetuses) exposed to ZIKV infection during pregnancy^17^. Suspected and probable cases were not included in the study.

### Data source

The non-nominal data from the RESP-Microcephaly were provided by the Data Governance Center of the Ministry of Health, which provided the following data: total reported cases, total confirmed cases, cases under investigation, probable, discarded, inconclusive and excluded cases, deaths and abortions. Data on the total number of live births for each year were extracted from the Brazilian Information System on Live Births (SINASC, https://datasus.saude.gov.br/nascidos-vivos-desde-1994). Socioeconomic data (illiteracy rate, Gini index, per capita income, garbage collection services, sanitation facilities, private households) were extracted from the Brazilian Geography and Statistics Institute (IBGE, https://www.ibge.gov.br/). Due to the COVID-19 pandemic and the suspension of the Demographic Census in 2020, the socioeconomic data refer to the 2010 Demographic Census (https://censo2010.ibge.gov.br/).

### Data analysis

Frequency distribution tables and graphs were used to describe the variables under study. For the presentation of data in graphs and frequency distribution tables, some measures were grouped according to Brazilian regions.

Crude incidence and CZS-specific mortality rates were the measures of disease and death frequency used in this study, respectively. Disease burden was expressed through DALYs, as proposed by Murray et al.^15^. Initially, years of life lost due to premature death (YLL) and years lived with severe disability (YLD) were calculated^15^. The average life expectancy at birth used in the study was 76.6 years^18^, as the sex of the cases was not taken into account. To calculate the YLD, the value of the weight of the disability and the duration of the disease are necessary. Up to the time at which this study was carried out, the value of weight/burden of disability for CZS and the duration of the disease had not been described in the literature. For this reason, we used the value adopted by Mora-Salamanca et al.^19^ for calculating this metric when estimating the DALYs for ZIKV-associated microcephaly in Colombia, which is 0.16. This value was described by Salomon et al.^20^ and Alfaro-Murillo et al.^12^ and refers to the disability weight for severe intellectual disability. For the duration of the disease, we used the value of 35 years, which was proposed by Honeycutt et al.^21^ and also refers to the duration of intellectual disability (mental retardation) and was used in the calculation of DALYs in Colombia^19^.

Associations between the health indicators estimated in the study (incidence, specific mortality and DALYs) and the socioeconomic variables were evaluated using Spearman's correlation coefficient, since the variables do not have a normal distribution of probabilities (verified by the Shapiro–Wilk test).

The analyses were performed using the R software version 4.0.4, considering a significance level of 5% (α = 0.05).

### Spatial analysis

For DALYs representation, as well as incidence and mortality rate, choropleth maps were constructed in ArcGis software version 10.6.1. with separatrices in quartiles. Spatial autocorrelation was verified, which analyzes the structure of dependence between the values observed in various areas; that is, it measures the correlation of the variable itself with space. In order to understand such aspects, it was decided to apply the Local Bivariate Moran Index, which indicates the degree of association (positive or negative) between the value of a variable in a given region with that of another variable in the same region. Based on a matrix of neighborhood, the measure of this correlation can have a value from 1 to − 1; the closer to one, the greater the similarity of the neighbors, while the inexistence of correlation for null values and negative values indicate dissimilarities between the neighbors^22^.

With this, it is possible to map the degree of association through statistically significant values in choropleth maps. The interpretation is based on the identification of high–high areas (high values of the first variable with high values of the second variable), low–low areas (low in the first and second variables), high–low areas (high in the first and low in the second variable) and low–high areas (low in the first and high in the second variable)^23^.

For this study, the neighborhood matrix by contiguity of the Queen type was considered due to the nature of the event and as the first variable, we chose to prioritize the DALYs, sequenced by the literacy rate, Gini index, and per capita income.

### Ethical aspects

This study was fully based on secondary data in the public domain that are made available in the official information systems of the Ministry of Health and the IBGE; therefore, it does not require ethical consideration.

## Results

**Figure 1:**
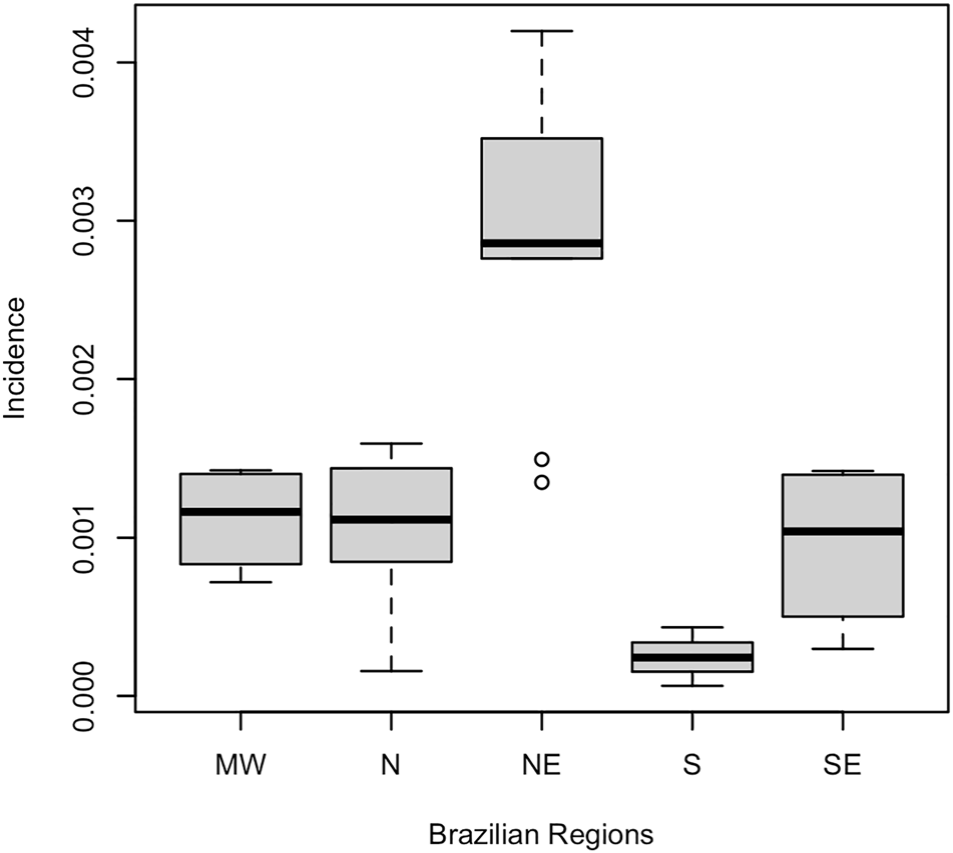
Boxplot for CZS incidence according to Brazilian regions, 2015–2020.

From 2015 to 2020, 3591 confirmed cases of CZS were reported in Brazil (cumulative incidence of 44.03 cases per 1000 live births). Table 1 presents the means of the incidence of CZS according to Brazilian regions, including standard deviations for the accumulated period 2015–2020, while Fig. 1 illustrates the variability of this measure between Brazilian regions. There is a great variability of incidence between regions, with emphasis on the low variability between the Midwest, Southeast and North regions, which contrasts with the South and Northeast regions. The presence of outliers in the Northeast region is justified by the two states (Ceará and Alagoas) where the lowest incidence occurred compared to the other states in the region.

**Table 1:** Mean of frequency measures for disease, death and burden of disease (DALY) by SCZ according to regions of Brazil, 2015–2020.

Region	Number of states per region	Incidence per 1000 live births		Mortality rate per 1000 live births		DALYs (years)	
		Mean	SD	Mean	SD	Mean	SD
Midwest	4*	1.12	0.34	0.09	0.03	421.31	261.64
Northeast	9	2.92	0.98	0.34	0.20	2110.77	1394.72
North	7	1.06	0.52	0.26	0.16	459.61	229.80
Southeast	4	0.95	0.54	0.11	0.10	1340.51	536.20
South	3	0.24	0.19	0.03	0.01	255.33	44.23

*3 states and 1 Federal District; SD, standard deviation.

When analyzing the time series year by year, the incidence of CZS was higher in 2016 with 24.15 cases per 1000 live births and lower in 2020 with 0.41 cases per 1000 live births. The Northeast region had the highest incidences per 1000 live births in 2015 (9.73), 2016 (12.59), 2017 (1.54), 2018 (1.27) and 2019 (0.61). In 2020, the North region had the highest incidence with 0.12 cases per 1000 live births. The regions least affected by CZS varied within the time series evaluated. In 2015, 2016 and 2017, the South region had the lowest incidence with 0.012, 0.3 and 0.14 cases per 1000 live births, respectively. In 2018, the Midwest, North and South regions showed the same results, with 0.18 cases per 1000 live births. In 2019, the Southeast region was the least affected with an estimated 0.06 cases per 1000 live births. In 2020, the Southeast and South regions were the least affected with 0.07 and 0 cases per 1000 live births, respectively. The details of incidence values by year and state are provided in Supplementary Table S1.

**Figure 2:**
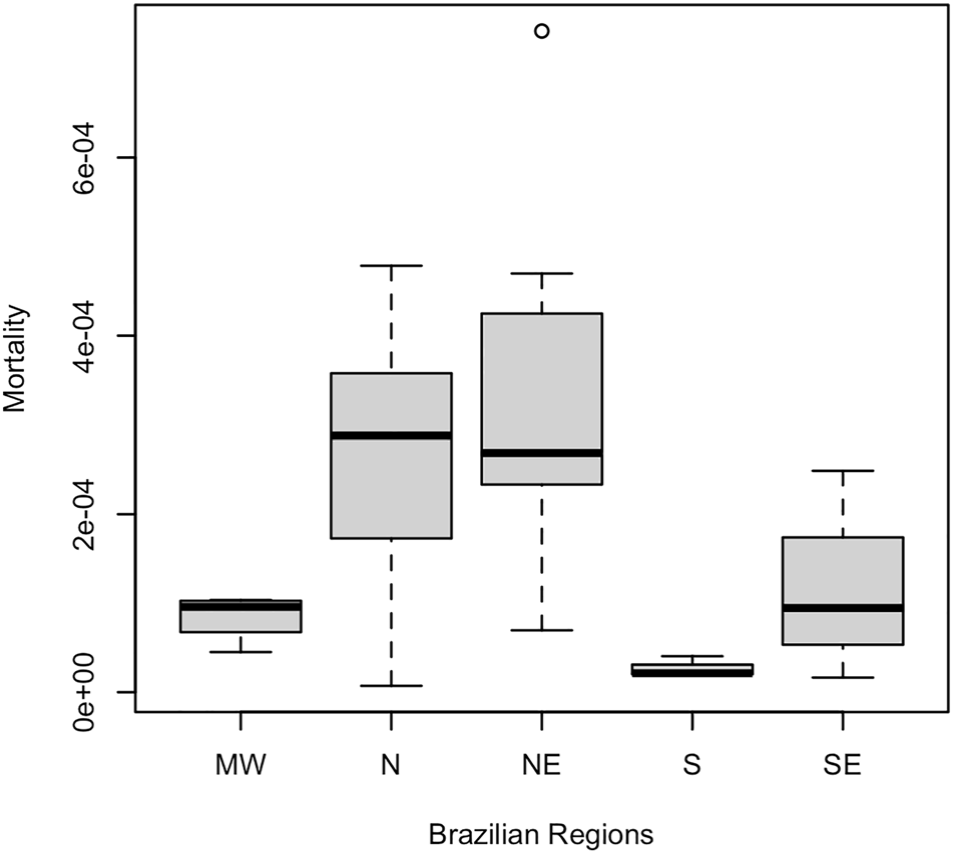
Boxplot for CZS-specific mortality rate according to Brazilian regions, 2015–2020.

The cumulative CZS specific mortality rate was 12.35 deaths per 1000 live births. Table 1 presents the mean values of the mortality rate for CZS according to regions and their respective standard deviations for the accumulated period 2015–2020. Figure 2 illustrates the variability of this measure across regions. There is high variability between regions and an outlier in the Northeast region due to a state (Rio Grande do Norte) that presented a mortality rate above the average of the other states in the region.

When analyzing the time series of the annual mortality rate, variability stands out between the states that were most affected, with the Northeast region having the highest results in 2015 (1.11 deaths per 1000 live births), 2018 (0.15 deaths per 1000 live births), 2019 (0.11 deaths per 1000 live births) and 2020 (0.01 deaths per 1000 live births). The North region was the most affected in 2016 (1.48 deaths per 1000 live births) and 2017 (0.55 deaths per 1000 live births). The South region was the least affected in all years. All estimates for each state and year are presented in Supplementary Table S1.

**Figure 3:**
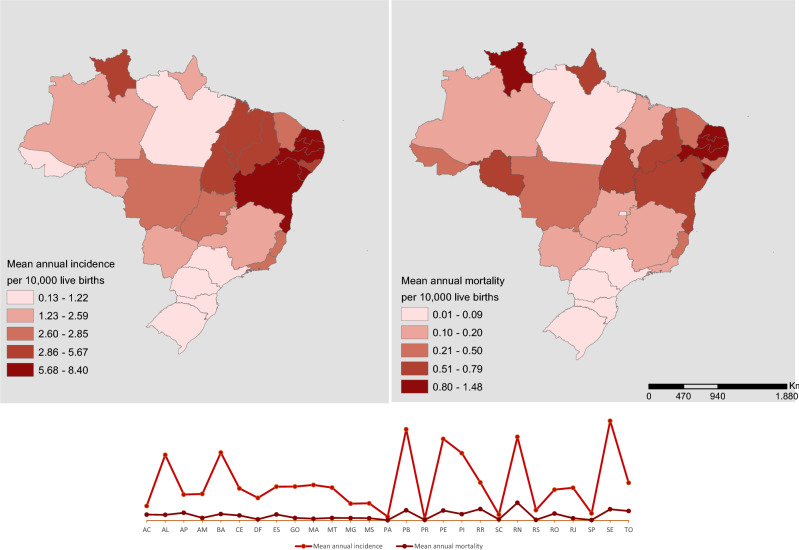
Spatial distribution of incidence and specific mortality rate by CZS according to Brazilian states, 2015–2020.

Figure 3 illustrates the spatial distribution of the incidence and specific mortality rate by CZS according to Brazilian states showing that the states of the Northeast, Southeast and Central-West regions have the highest rates.

**Figure 4:**
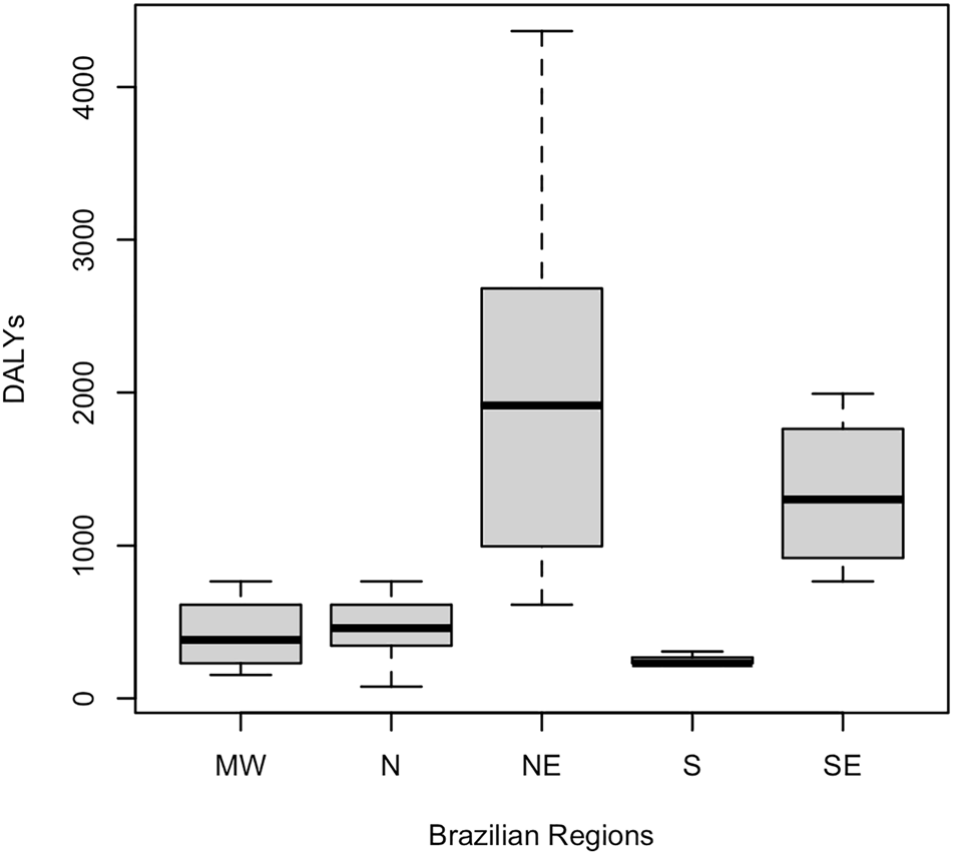
DALYs boxplot according to Brazilian regions, 2015–2020.

A loss of 30,027.44 DALYs was estimated in the period from 2015 to 2020. The YLL had a value of 30,027.2 and the YLD 0.24 of the total value of the DALY. The year 2016 recorded the highest amount of DALYs lost, with a loss of 16,239.33 DALYs. In 2020, the lowest loss occurred, with 229.8 DALYs (Supplementary Table S1). Table 1 presents the DALY measurements for CZS and their respective standard deviations for the accumulated period 2015–2020 and Fig. 4 illustrates the variability of these measurements for the regions. It is possible to observe that there is a very significant variation when we compare the Northeast region with the other regions, mainly in relation to the South region, which presented lower values, less dispersion and greater homogeneity. The Midwest and North regions showed similar variability and dispersion.

During the 6 years of analysis, the Northeast region had the highest values of lost DALYs. During the years 2016 and 2017, Bahia presented the highest estimates of DALYs lost with 2757.60 and 459.60, respectively. In 2015, Pernambuco was the state with the highest value, with 2,221.41 DALYs lost, while in 2018 it was the state of Paraíba, with 383 DALYs loss. In 2019 and 2020, the state that presented the highest estimated DALYs was Minas Gerais, which is in the Southeast region, with 306.4 and 76.6, respectively.

**Figure 5:**
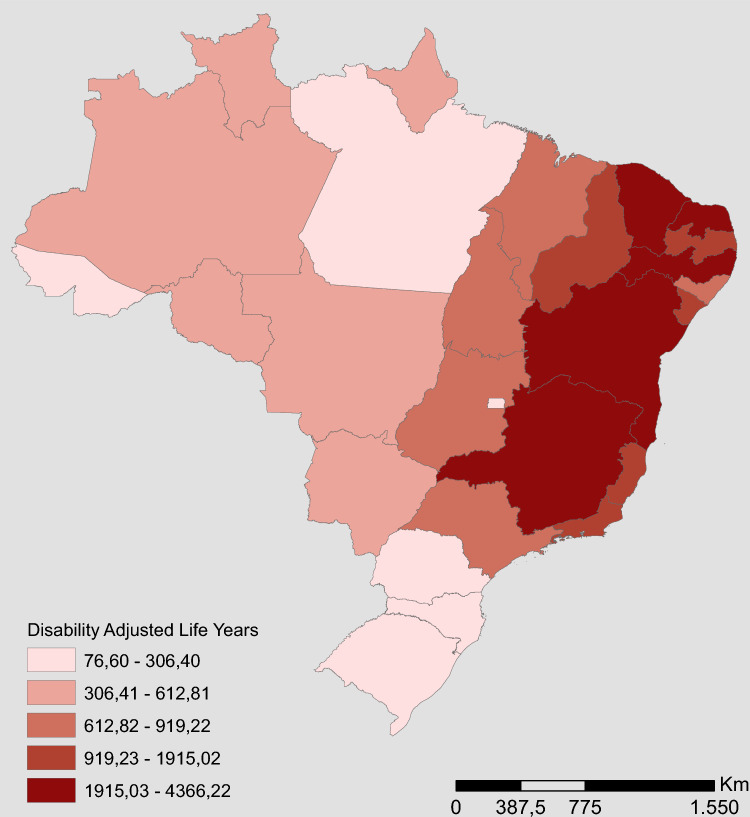
Spatial distribution of DALYs for CZS, Brazil, 2015–2020.

Figure 5 illustrates the spatial distribution of DALY for CZS, which presents a distribution pattern very similar to that observed for specific incidence and mortality, which are plotted in Fig. 3.

**Figure 6:**
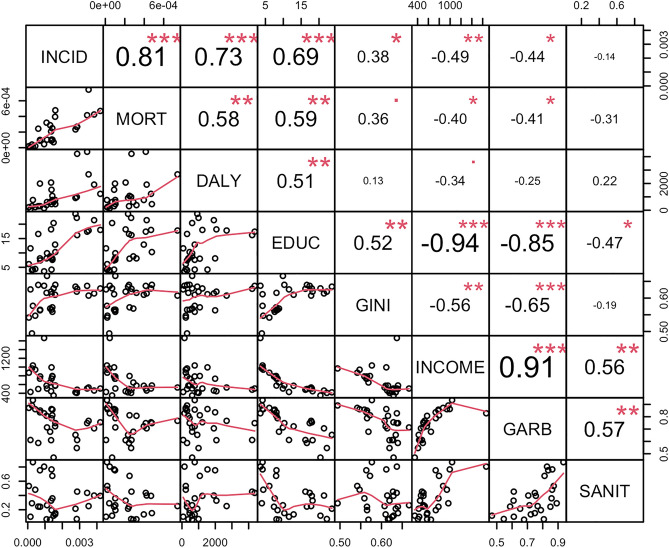
Correlation and dispersion matrix for CZS and socioeconomic indicators. *INCID* incidence of CZS from 2015 to 2020, *MORT* CZS-specific mortality rate of CZS from 2015 to 2020, *DALY* disability-adjusted life years lost for the period 2015–2020, *EDUC* illiteracy rate; GINI: Gini index, *INCOME* per capita income, *GARB* proportion of households with regular garbage collection, *SANIT* proportion of households with piped sewage. ***p < 0.001; **p < 0.05; *p = 0.10.

The correlation analysis between the health indicators for CZS and the socioeconomic indicators are presented in Table 2 and Fig. 6, which shows the correlation and dispersion matrix for these analyses. The incidence of CZS showed a significant correlation with the Gini index (ρ = 0.38; p-value = 0.04), per capita income (ρ = − 0.49; p-value < 0.01) and data collection garbage per cleaning service (ρ = − 0.44; p-value = 0.02). Considering the specific mortality by CZS, significant correlations were observed with the illiteracy rate (ρ = 0.59; p-value < 0.01), per capita income (ρ = − 0.40; p-value < 0.01) and garbage collection by cleaning service (ρ = − 0.40; p-value = 0.03). DALY showed a significant correlation with the illiteracy rate (ρ = 0.50; p-value < 0.01) and with the proportion of households that have a sanitary installation by septic tank and absence of piped sewage (ρ = 0.47; p-value = 0.01).

**Table 2:** Correlation between health and socioeconomic indicators.

Socioeconomic indicators	Incidence		Mortality		DALY	
	ρ	p-value	ρ	p-value	ρ	p-value
Illiteracy rate	0.69	< 0.01	0.59	< 0.01	0.51	< 0.01
Gini index	0.38	0.04	0.36	0.06	0.13	0.51
Per capita income	− 0.49	< 0.01	− 0.40	0.03	− 0.34	0.08
Garbage collection service (collection by cleaning service)	− 0.44	0.02	− 0.41	0.03	− 0.25	0.20
Sanitary installation (piped sewage)	− 0.14	0.46	− 0.31	0.12	0.22	0.26

No significant correlations were observed between the CZS measures and the socioeconomic indicator proportion of households with piped sewage.

These results suggest the existence of a positive association between the illiteracy rate and all health indicators evaluated. Considering that values of the Gini index close to 1 indicate high income inequality in the territory, the results of this study suggest the existence of a direct relationship between income inequality and the incidence of CZS. Moreover, the results of correlations between CZS indicators and per capita income suggest the existence of an inversely proportional association.

**Figure 7:**
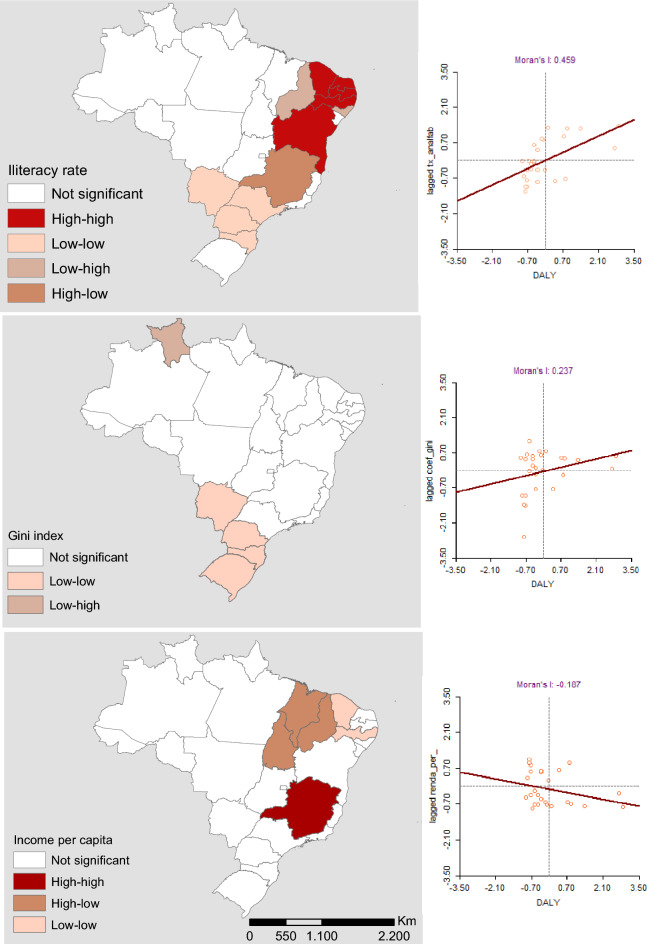
Spatial autocorrelation between DALYs and literacy rate, Gini Index, and per capita income according to neighborhood, Brazil, 2022.

When evaluating the Local Bivariate Moran Index, it was possible to identify a spatial autocorrelation between the Brazilian regions with the greatest similarity between the DALY and the literacy rate (Moran'I = 0.45), followed by the Gini Index (Moran'I = 0.23) and dissimilarity between DALY and per capita income (Moran'I = − 0.18). Figure 7 represents the areas with correlation of variables and their surroundings.

## Discussion

In March 2015, the Brazilian surveillance system detected the first case of ZIKV fever in Brazil^24^, while the first possible cases of microcephaly associated with ZIKV in Brazil were recorded in October of the same year^25,26^; since then, several studies have been carried out to improve the understanding of events related to ZIKV infection^27,28^. However, there are still few studies that have investigated the magnitude and burden of CZS or of microcephaly related to the ZIKV^29^, as well as the potential socioeconomic factors associated with the disease. Our study demonstrated a considerable loss of 30,027.44 DALYs in the accumulated period of six years evaluated in the study, in addition to demonstrating the correlation of the disease with socioeconomic indicators, especially schooling.

The ZIKV epidemic remains a serious public health problem for children with CZS. Despite the sharp drop in cases since 2018, Brazil, as a continental country with a predominantly tropical climate, has environmental conditions favorable to the occurrence of new epidemics caused by arboviruses such as ZIKV^30^.

The number of live births with microcephaly in Brazil increased considerably in 2015, a number which was nine times greater than those recorded in the period from 2000 to 2014^31^. This peak was also observed in several other countries during the epidemic, such as Colombia^32^, the United States^33^, French Polynesia^34^ and Costa Rica^35^, which had a four-fold increase in the prevalence of microcephaly cases.

A previous report that documented the regional patterns in the spatial distribution of microcephaly risk related to the ZIKV in Brazil showed high spatial heterogeneity between regions and highlighted the Northeast region as the area most disproportionately affected area^29^, which is consistent with our results. These findings reinforce the data reported by Marinho et al.^36^, in that the Northeast region has the highest disease burden for several diseases, including infectious ones. Contrarily, the state of Rio Grande do Sul, located in southern Brazil, experienced minimal impact from the epidemic. Contrarily, the state of Rio Grande do Sul, located in southern Brazil, experienced minimal impact from the epidemic. This disparity can be attributed to the region's climate, characterized by the lowest temperatures in Brazil, which creates an unfavorable environment for the proliferation and persistence of the mosquito vector responsible for transmitting certain arboviruses^31,37^.

As already demonstrated in previous studies, the peak of CZS cases occurred in 2016, with a rapid reduction in incidence in the following years^5,38^. We can associate this decrease in cases with the control and prevention actions against ZIKV that were applied mainly in the control of *Ae. aegypti*, the main vector of the virus^5,17,30^, which resulted in a decrease not only in cases of Zika fever but also in cases of CZS over the years. The cyclic behavior of the incidence of some arboviruses should also be considered^39–41^, which may also explain the decrease in ZIKV viral circulation in Brazil. Despite the drop in notifications of new cases of ZIKV infections and CZS cases, 5,699 probable cases of ZIKV infection were reported between January and June 2022 in Brazil^16^.

The CZS-specific mortality rate was higher in 2016 in all regions, especially in the Northeast region, which also had a higher incidence and lost DALYs. During the six years observed in this study, it was noted that mortality had a significant drop from the year 2018. The year 2017 also had a high mortality number, but this was still low when compared to 2016.

No specific studies for the burden of disease by CZS were found in the literature, but there were two studies on microcephaly associated with ZIKV^12,19^. More-Salamanca et al.^19^, estimated 9.48 DALYs lost for each case of microcephaly associated with Zika virus in Colombia from 2015 to 2016, a value that is higher than that estimated in this study, which was 8.36 DALYs lost for each case from 2015 to 2020. When considering the period from 2015 to 2016, our analysis reveals that the estimated DALYs lost in Brazil are lower than the number of DALYs lost in Colombia. This difference can be attributed to the fact that our study focused solely on confirmed cases of CZS, while the study by More-Salamanca et al.^19^ included both probable and confirmed cases of microcephaly associated with the ZIKV. The inclusion of probable cases in their analysis likely contributed to a higher overall burden of disease in Colombia compared to our analysis of confirmed CZS cases in Brazil. It is important to consider these distinctions when interpreting and comparing the results between the two studies.Alfaro-Murillo et al.^12^ also carried out a study on the burden of disease associated with microcephaly, as already discussed by More-Salamanca et al.^19^; the values estimated by these authors were very high, with 29.95 DALYs lost for each case of microcephaly. This difference may be due to the different parameters used for the DALYs estimative, with only the weight of the disability being the same used in both studies.

The 2015 CZS disease burden estimated in our study (7506.82 DALYs) was higher when compared to other congenital anomalies (in the same year) according to WHO global estimates (649.2 DALYs), with 59.7 DALYs attributable to breast and neural tube defects and 300 DALYs to other unspecified congenital anomalies^42^. For comparison purposes, the estimate of CZS in the year 2019 was 1072.4 DALYs, which is lower than that estimated by the WHO for congenital anomalies in the same year (1158.8 DALYs)^42^. Finally, based on the results obtained in this study, we can suggest that if CZS was considered in the burden of disease analyzes conducted by the WHO, it would be responsible for most of the value obtained.

It should be noted that ZIKV fever and, consequently, CZS are relatively new diseases and that our data refer only to the 6 years since the disease first occurred in Brazil. In this sense, the maximum age of children is 6 years, highlighting the need for future studies to verify survival and disease burden (in terms of DALY) for individuals affected by CZS.

ZIKV, like other arboviruses transmitted by *Ae. aegypti*, has a higher risk of transmission in tropical areas where basic sanitation conditions are poor and family income is low^43^. Considering that Brazil is a country with high social inequality, studies have shown that the lack of access to schooling, job opportunities and poverty is a determining factor when we refer to the ZIKV epidemic and children born with CZS in the country^44^. The fact that our results show that CZS is directly linked to illiteracy, low incomes and poor basic sanitation conditions confirm that the more vulnerable the family, the greater the chances of the mother being infected by ZIKV and, consequently, the risk of vertical transmission of ZIKV and the occurrence of CZS. During the 2015–2016 epidemic, descriptive studies reported that mothers of children with CZS had low schooling and were unemployed, especially after the birth of their children^45,46^.

Considering that the emergence of the CZS epidemic in Brazil began in 2015, our analysis included only children up to the age of six. Therefore, it is crucial to undertake comprehensive and longitudinal studies that delve into the development progress of children affected by CZS. Through such research endeavors, it becomes possible to gain valuable insights into the developmental trajectories of these children and the specific challenges faced by their families.

As one of the pioneering studies conducted in Brazil to assess the overall burden of CZS, our research provides valuable insights into the potential impact of the disease within the country. Moreover, our results strongly indicate that the collective efforts to combat the CZS epidemic in Brazil have resulted in a consistent decline in new cases of CZS since 2017. Due to access to all the results of reported and confirmed cases and deaths from CZS, it was possible to have a broad view of the situation of the disease in Brazil. Our results may also help to understand future studies. Although previous report have mapped the risk of microcephaly related to ZIKV in Brazil^29,44^, our study advances in the analysis of the association of socioeconomic factors with CZS, strengthening the hypothesis of the action of inequities in health as determinants of the health-disease process and the occurrence of CZS. An important limitation of our study is the use of the sociodemographic data from the 2010 census, as the 2020 Brazilian census was not conducted due to the COVID-19. However, it is worth noting that since our study analyzed CZS data since 2015, the data from the 2010 census might not have experienced sudden and significant changes during the intervening period. While this limitation affects the completeness of our analysis, we believe that it does not substantially impact the overall findings and conclusions of the study. Another limitation is that because CZS is a new disease, there is still no specific value of disability weight for CZS.

To date, this is the first study carried out to estimate the burden of disease from CZS carried out in Brazil and one of the few in South America. From our results, it was possible to observe that the Northeast region was responsible for the largest DALYs lost during 2015–2020 and the year 2016 had the largest loss among all years. Based on the weaknesses of our study, we see that there are still issues that can be resolved with future studies, such as assessing the quality and life expectancy of people born with CZS and also a study conducted to assess the weight of disability by CZS and to ZIKV. It is possible that our results and methodologies will help with the process in the new studies and for future comparisons.

## Supplementary Information

Supplementary Table S1.

## Data Availability

All relevant data that support the findings of this study are presented within the manuscript and its additional files.
