# Supplementary material for: Disease burden of congenital Zika virus syndrome in Brazil and its association with socioeconomic data
Source: Sci Rep. 2023 Jul 23;13:11882. doi: 10.1038/s41598-023-38553-4 (PMC10363536; doi:10.1038/s41598-023-38553-4)
Supplement: Supplementary file 1 — Supplementary Table S1. [file 41598_2023_38553_MOESM1_ESM.pdf]

Disease burden of congenital Zika virus syndrome in Brazil and its association with socioeconomic data

Bruna Luiza de Amorin Vilharba, Mellina Yamamura, Micael Viana de Azevedo, Wagner de Souza Fernandes, Cláudia Du Bocage Santos-Pinto, Everton Falcão de Oliveira

Supplementary Table S1. **Incidence, specific mortality rate, and DALYs for congenital Zika virus syndrome (CZS) according to year, region, and state, Brazil, 2015–2020**

REGIONS and states	Year	CZS cases	Incidence per 1000 live births	Mortality rate per 1000 live births	DALYs
**MIDWEST**	**2015**	**21**	**0.31**	**0.02**	**76.6**
Distrito Federal	2015	2	0.04	0	0
Goiás	2015	7	0.06	0	0
Mato Grosso do Sul	2015	1	0.02	0.02	76.6
Mato Grosso	2015	11	0.19	0	0.001

**NORTHEAST**	**2015**	**843**	**9.73**	**1.11**	**7,047.22**
Alagoas	2015	18	0.13	0.02	229.8
Bahia	2015	168	0.81	0.04	766
Ceará	2015	56	1.14	0.18	689.4
Maranhão	2015	31	0.88	0.02	76.6
Paraíba	2015	98	1.65	0.11	536.2
Pernambuco	2015	265	2.25	0.24	2,221.41
Piauí	2015	22	0.44	0.06	229.8
Rio Grande do Norte	2015	95	1.81	0.38	1,532.01
Sergipe	2015	90	0.62	0.06	766
**NORTH**	**2015**	**7**	**0.18**	**0.05**	**76.6**
Acre	2015	3	0.05	0.05	76.6
Amazonas	2015	80	0	0	0
Amapá	2015	5	0.01	0	0

Pará	2015	3	0.01	0	0
Rondônia	2015	43	0	0	0
Roraima	2015	29	0	0	0
Tocantins	2015	2	0.11	0	0
**SOUTHEAST**	**2015**	**1**	**0.31**	**0.02**	**306.4**
Espirito Santo	2015	1	0.08	0.01	76.6
Minas Gerais	2015	0	0.01	0	0
Rio de Janeiro	2015	3	0.18	0.01	229.8
São Paulo	2015	80	0.04	0	0
**SOUTH**	**2015**	**5**	**0.012**	**0**	**0**
Paraná	2015	3	0.006	0	0
Rio Grande do Sul	2015	43	0.006	0	0
Santa Catarina	2015	29	0	0	0
**TOTAL**	**2015**	**953**	**10.54**	**1.2**	**7,506.82**

**MIDWEST**	**2016**	**191**	**3.09**	**0.29**	**1,378.81**
Distrito Federal	2016	16	0.36	0.04	153.2
Goiás	2016	80	0.83	0.07	536.2
Mato Grosso do Sul	2016	28	0.65	0.07	229.8
Mato Grosso	2016	67	1.25	0.11	459.6
**NORTHEAST**	**2016**	**1.081**	**12.59**	**1.31**	**8,709.07**
Alagoas	2016	80	1.66	0.10	383
Bahia	2016	340	1.70	0.18	2,757.6
Ceará	2016	108	0.85	0.13	1,302.2
Maranhão	2016	109	0.98	0	0
Paraíba	2016	97	1.72	0.16	689.4
Pernambuco	2016	160	1.22	0.17	1,761.8
Piauí	2016	83	1.76	0.17	612.8
Rio Grande do Norte	2016	57	1.25	0.28	995.8

Sergipe	2016	47	1.45	0.12	306.4
**NORTH**	**2016**	**149**	**5.14**	**1.48**	**2,144.83**
Acre	2016	9	0.57	0.19	229.8
Amazonas	2016	44	0.57	0.02	153.2
Amapá	2016	11	0.70	0.32	383
Pará	2016	17	0.12	0.007	76.6
Rondônia	2016	20	0.75	0.18	383
Roraima	2016	17	1.14	0.52	459.6
Tocantins	2016	31	1.29	0.25	459.6
**SOUTHEAST**	**2016**	**478**	**2.54**	**0.29**	**3,600.21**
Espirito Santo	2016	52	0.97	0.16	689.4
Minas Gerais	2016	93	0.36	0.06	1,302.2
Rio de Janeiro	2016	230	1.04	0.06	1,072.4
São Paulo	2016	103	0.17	0.01	536.2

**SOUTH**	**2016**	**41**	**0.3**	**0.09**	**306.4**
Paraná	2016	7	0.04	0.01	153.2
Rio Grande do Sul	2016	26	0.18	0.007	76.6
Santa Catarina	2016	8	0.08	0.08	76.6
**TOTAL**	**2016**	**1.940**	**23.66**	**3.46**	**16,139.32**
**MIDWEST**	**2017**	**46**	**0.66**	**0**	**0**
Distrito Federal	2017	15	0.33	0	0
Goiás	2017	28	0.28	0	0
Mato Grosso do Sul	2017	2	0.03	0	0
Mato Grosso	2017	1	0.02	0	0
**NORTHEAST**	**2017**	**138**	**1.54**	**0.14**	**1,225.6**
Alagoas	2017	21	0.41	0.03	153.2
Bahia	2017	49	0.24	0.02	459.6
Ceará	2017	3	0.02	0	0

Maranhão	2017	14	0.12	0.02	229.8
Paraíba	2017	8	0.13	0.03	153.2
Pernambuco	2017	21	0.15	0.007	76.6
Piauí	2017	9	0.18	0	0
Rio Grande do Norte	2017	10	0.21	0.04	153.2
Sergipe	2017	3	0.08	0	0
**NORTH**	**2017**	**53**	**1.5**	**0.55**	**842.61**
Acre	2017	0	0	0	0
Amazonas	2017	25	0.32	0.32	383
Amapá	2017	5	0.32	0	0
Pará	2017	3	0.02	0	0
Rondônia	2017	13	0.47	0.07	153.2
Roraima	2017	2	0.17	0	0
Tocantins	2017	5	0.20	0.16	306.4

**SOUTHEAST**	**2017**	**107**	**0.56**	**0.07**	**766**
Espirito Santo	2017	16	0.28	0.05	229.8
Minas Gerais	2017	39	0.14	0.01	229.8
Rio de Janeiro	2017	24	0.10	0.008	153.2
São Paulo	2017	28	0.04	0.003	153.2
**SOUTH**	**2017**	**18**	**0.14**	**0.006**	**229.8**
Paraná	2017	1	0.006	0	76.6
Rio Grande do Sul	2017	10	0.07	0.02	0
Santa Catarina	2017	7	0.07	0.02	153.2
**TOTAL**	**2017**	**362**	**4.4**	**0.78**	**3,064.03**
**MIDWEST**	**2018**	**16**	**0.18**	**0.03**	**229.8**
Distrito Federal	2018	1	0.02	0	0
Goiás	2018	14	0.14	0.03	229.8
Mato Grosso do Sul	2018	1	0.02	0	0

Mato Grosso	2018	0	0	0	0
**NORTHEAST**	**2018**	**100**	**1.27**	**0.15**	**1,149**
Alagoas	2018	22	0.41	0.01	76.6
Bahia	2018	14	0.06	0.009	153.2
Ceará	2018	5	0.03	0.007	76.6
Maranhão	2018	10	0.08	0.02	229.8
Paraíba	2018	16	0.26	0.08	383.0
Pernambuco	2018	18	0.13	0.01	153.2
Piauí	2018	12	0.24	0	0
Rio Grande do Norte	2018	2	0.04	0	0
Sergipe	2018	1	0.02	0.02	76.6
**NORTH**	**2018**	**10**	**0.18**	**0.03**	**76.6**
Acre	2018	7	0	0	0
Amazonas	2018	0	0.08	0	0

Amapá	2018	0	0	0	0
Pará	2018	1	0	0	0
Rondônia	2018	1	0.003	0.03	76.6
Roraima	2018	1	0.07	0	0
Tocantins	2018	43	0.03	0	0
**SOUTHEAST**	**2018**	**7**	**0.22**	**0.01**	**306.4**
Espirito Santo	2018	14	0.12	0.01	76.6
Minas Gerais	2018	8	0.05	0.003	76.6
Rio de Janeiro	2018	14	0.03	0.004	76.6
São Paulo	2018	24	0.02	0.001	76.6
**SOUTH**	**2018**	**1**	**0.18**	**0.01**	**153.2**
Paraná	2018	17	0.006	0	0
Rio Grande do Sul	2018	6	0.12	0.01	153.2
Santa Catarina	2018	193	0.06	0	0

**TOTAL**	**2018**	**16**	**2.03**	**0.23**	**1,915.01**
**MIDWEST**	**2019**	**14**	**0.20**	**0**	**0**
Distrito Federal	2019	5	0.11	0	0
Goiás	2019	8	0.08	0	0
Mato Grosso do Sul	2019	0	0	0	0
Mato Grosso	2019	1	0.01	0	0
**NORTHEAST**	**2019**	**50**	**0.61**	**0.11**	**612.8**
Alagoas	2019	1	0.02	0.02	76.6
Bahia	2019	13	0.06	0.002	76.6
Ceará	2019	3	0.02	0	0
Maranhão	2019	6	0.05	0	0
Paraíba	2019	4	0.06	0.01	76.6
Pernambuco	2019	7	0.05	0.01	153.2
Piauí	2019	12	0.25	0.04	153.2

Rio Grande do Norte	2019	2	0.04	0	0
Sergipe	2019	2	0.06	0.03	76.6
**NORTH**	**2019**	**6**	**0.09**	**0.01**	**76.6**
Acre	2019	0	0	0	0
Amazonas	2019	5	0.06	0.01	76.6
Amapá	2019	0	0	0	0
Pará	2019	0	0	0	0
Rondônia	2019	1	0.03	0	0
Roraima	2019	0	0	0	0
Tocantins	2019	0	0	0	0
**SOUTHEAST**	**2019**	**22**	**0.06**	**0.01**	**306.4**
Espirito Santo	2019	0	0	0	0
Minas Gerais	2019	17	0.06	0.01	306.4
Rio de Janeiro	2019	0	0	0	0

São Paulo	2019	5	0.008	0	0
**SOUTH**	**2019**	**10**	**0.08**	**0.01**	**76.6**
Paraná	2019	0	0	0	0
Rio Grande do Sul	2019	7	0.05	0	0
Santa Catarina	2019	3	0.03	0.01	76.6
**TOTAL**	**2019**	**102**	**1.04**	**0.14**	**1,072.4**
**MIDWEST**	**2020**	**6**	**0.10**	**0**	**0**
Distrito Federal	2020	3	0.07	0	0
Goiás	2020	3	0.03	0	0
Mato Grosso do Sul	2020	0	0	0	0
Mato Grosso	2020	0	0	0	0
**NORTHEAST**	**2020**	**7**	**0.08**	**0.01**	**153.2**
Alagoas	2020	0	0	0	0
Bahia	2020	1	0.005	0	0

Ceará	2020	0	0	0	0
Maranhão	2020	2	0.01	0.009	76.6
Paraíba	2020	3	0.05	0.01	76.6
Pernambuco	2020	0	0	0	0
Piauí	2020	1	0.02	0	0
Rio Grande do Norte	2020	0	0	0	0
Sergipe	2020	0	0	0	0
**NORTH**	**2020**	**7**	**0.12**	**0**	**0**
Acre	2020	0	0	0	0
Amazonas	2020	6	0.08	0	0
Amapá	2020	0	0	0	0
Pará	2020	0	0	0	0
Rondônia	2020	1	0.04	0	0
Roraima	2020	0	0	0	0

Tocantins	2020	0	0	0	0
**SOUTHEAST**	**2020**	**20**	**0.07**	**0.004**	**76.6**
Espirito Santo	2020	4	0	0	0
Minas Gerais	2020	10	0.07	0.004	76.6
Rio de Janeiro	2020	0	0	0	0
São Paulo	2020	6	0.003	0	0
**SOUTH**	**2020**	**1**	**0**	**0**	**0**
Paraná	2020	0	0	0	0
Rio Grande do Sul	2020	1	0	0	0
Santa Catarina	2020	0	0	0	0
**TOTAL**	**2020**	**53**	**0.37**	**0.01**	**229.8**
